# Role of private providers in the implementation of the national health insurance scheme in Zambia: a qualitative study of perceptions and experiences

**DOI:** 10.1136/bmjopen-2024-092047

**Published:** 2025-02-10

**Authors:** Warren Mukelabai Simangolwa, Jesper Sundewall

**Affiliations:** 1 Centre for Health Economics, Financing and Technology Assessment, Patient and Citizen Involvement in Health, Lusaka, Zambia; 2 Health Economics and HIV/AIDS Research Division, University of KwaZulu-Natal, Durban, South Africa; 3 Department of Clinical Sciences, Lund University, Malmö, Sweden

**Keywords:** Health policy, Accreditation, QUALITATIVE RESEARCH, Health Services Accessibility, HEALTH ECONOMICS, Quality in healthcare

## Abstract

**Abstract:**

**Introduction:**

An increasing number of sub-Saharan African countries are implementing national health insurance schemes (NHISs) to support the aspiration of universal health coverage (UHC). A growing body of literature recognises the private sector role in improving NHIS equity in service access, public provider complementarity and overall member satisfaction. Zambia has implemented the NHIS since 2019 as a priority health financing strategy to support UHC. This study provides the first examination of the private sector’s experiences implementing the Zambia NHIS.

**Methods:**

The study uses a qualitative study design and inductively synthesises data from 30 in-depth interviews with the private sector accredited to the Zambia NHIS in one rural and one urban province.

**Results:**

The private sector was motivated by profits and complementarity with public providers regarding service readiness and availability. Providers perceived the accreditation process and fees as standard and affordable. Providers reported increased service utilisation, mainly by the NHIS clients. Senior citizens and the informal sector constituted the majority of users. There were implementation challenges, including delays in preauthorisations, loss of member details and exclusion and restrictions of interventions in the benefits package. Private providers also experienced overcrowding and reduced service quality. Providers perceived the service reimbursement levels as relatively cost-reflective, but some providers shifted models to maximise profits, including inducing demand to defraud the fund. Generally, providers perceived payments as within the agreed timelines, especially for online claims.

**Conclusion:**

The initial scepticism among private providers before the initiation of the NHIS has subsided. The private provider’s experiences with accreditation, service utilisation, claims and reimbursements have been overall positive.

**STRENGTHS AND LIMITATIONS OF THIS STUDY**

- The novelty of this study is that it systematically analyses the experiences of private healthcare providers in implementing the National Health Insurance Scheme (NHIS) in Zambia as a follow-up to perceptions synthesised before the initiation of the NHIS.
- Responses were collected from facility managers in selected rural and urban provinces across various private sector facilities, including hospitals, clinics, pharmacies, dentists, opticians and laboratories, to incorporate diverse experiences and feedback.
- As the implementation of the NHIS is ongoing, the study attempted to capture these developments after the study period and discuss their relevance to the findings.
- This study was limited by focusing solely on the private sector and distributing the sample across various providers, potentially reducing the detail synthesised in each private-sector category.
- The findings from this study will contribute to the effective implementation of the NHIS to advance universal health coverage by providing evidence regarding the motivations, experiences and challenges private healthcare providers face in providing the benefits package.

## Introduction

 The aspiration of universal health coverage (UHC) means moving towards all people having access to quality health services without experiencing financial hardship.^1^ Increased out-of-pocket spending and stalled health service coverage have derailed global progress towards UHC by 2030, particularly in low- and middle-income countries (LMICs).^2^ The 2010 World Health Report recommended that countries reduce reliance on patient direct payments by introducing compulsory prepayments and pooling mechanisms.^1^ Although with varying terminology, LMICs use the National Health Insurance Scheme (NHIS) as a priority health financing strategy to pursue UHC.^3 4^ In sub-Saharan Africa, many countries have already implemented their NHIS, with others currently undergoing processes to initiate such schemes.^5 6^ Results from NHIS implementation in LMICs have shown some impact on improving financial risk protection.^7 8^

Implementing an NHIS requires critical reflections on the strategic purchasing function, including decisions on private provider participation.^9^ However, including the private sector requires that the government has sufficient regulatory capacity and has defined how private providers will complement public providers.^10^ Montagu and Goodman argue that developing a mutually beneficial contracting model is necessary for successfully engaging private providers.^11^ One example is Ghana, which revised its provider payment mechanism from a fee-for-service to diagnostic-related groupings to attract the private sector, which perceived the former reimbursement system as having lower tariffs.^12^ Private providers have argued that they can contribute to improved health equity by reaching underserved communities.^13^ Furthermore, private providers complement public providers by taking in more insured clients and enhancing patient choice, promptness in receiving care and overall satisfaction.^414^,^16^

There are also examples of less successful inclusion of private providers in insurance schemes. After 30 years of implementing health insurance in South Korea, there were concerns about the growing dominance of private for-profit providers, resulting in challenges to enforce government regulation, poor cost containment and long-run fiscal sustainability.^17^ Despite the available policy and regulatory frameworks for private providers, Ethiopia and Pakistan have yet to be able to use private providers to deliver the essential package of health services due to restrictions on service delivery by private providers to selected cities and a lack of transparency in contracting.^18^ In Ghana and Nigeria, members have reported poor quality and client experiences with private provider services.^14 19^ The Zambia NHIS was assented into law through Act 2 of 2018.^20^ This act established the National Health Insurance Management Authority (NHIMA), a quasi-government corporate body to manage the scheme.^20^ The Zambia NHIS implementation was initiated in 2019, followed by an initial 4-month waiting period to support revenue collection before service provision in February 2020.^21 22^ A study on private-sector inclusion conducted before the initiation of Zambia NHIS revealed a cautious willingness among private providers to join the scheme. Private providers cited their complementarity with public providers in the availability of medical equipment for diagnostics and laboratory testing, medications, healthcare services, timely service delivery and consistency in care standards.^21^ However, their hesitation to join Zambia NHIS included concerns about perceived high accreditation costs, lengthier claim processes, inflexible payment mechanisms and related payment delays.^21^ In another study conducted in the initial phases of Zambia NHIS implementation, stakeholders perceived, including private providers as necessary to support their integration into the health system, increase member choices and decongest public facilities.^23^ At the same time, stakeholders were concerned about how private providers would fill public service gaps, interact with the public referral system and support equity in service access for rural members.^23^

The Zambia NHIS is now in its fourth year of operation, and an increasing number of different categories of private providers have been accredited and become a part of the scheme. However, studies have yet to be conducted on the experiences of private providers as a part of implementing NHIS in Zambia. Thus, the purpose of this study is to explore private provider experiences in accreditation, service provision, claims and reimbursements in the Zambia NHIS to understand the status of the sector’s initial concerns.

## Methods

We conducted a qualitative study of private healthcare providers in the Southern and Lusaka provinces of Zambia, using semistructured interviews for data collection. The study builds on previous research on factors influencing private-sector inclusion in the NHIS, in which both authors participated.^21^ This study follows 21 reporting items of the Standards for Reporting Qualitative Research to increase transparency, trustworthiness and robustness in conducting, analysing and publishing qualitative research (online supplemental file 1).^24^

### Study context

In Zambia, the health system includes public, private and not-for-profit providers. Public facilities range from community-level primary healthcare to hospital services with increasing specialisation. In contrast, private and not-for-profit providers typically have fragmented services, including standalone hospitals, specialised clinics, laboratories, diagnostic centres, opticians and pharmacies. According to the Zambia National Health Strategic Plan 2022–2026, there are 2810 government facilities, while private and faith-based facilities account for 381 and 105, respectively.^25^ The Zambia NHIS has a mixed healthcare provider system, where public and private providers are accredited to provide hospital-level insured services. The private sector offers hospitals, hospices, dental clinics, pharmacies, laboratories and diagnostic and optician services to the scheme. The National Health Strategic Plan 2022–2026 estimates NHIS principal membership at 1 277 960.^25^

The NHIS was introduced as a domestic health financing mechanism to support Zambia’s attainment of UHC.^26^ Global health expenditure data estimate Zambia’s 2022 current health expenditure (CHE) at US$76 per capita, with the domestic general government expenditure constituting 41.4% of CHE. In the same year, the proportion of out-of-pocket spending increased to 9.7% from 8.1% in 2020, while national health insurance contributions accounted for 6.3% of CHE, an increase from 3.5% in 2020. Furthermore, external aid reached 41.4% in 2022 from 33% in 2020.^27^

### Study participants

The study used the national health strategic plan definition of the private sector, which separates the sector from not-for-profit, public and mine provider facilities.^28^ 180 private providers, representing 54.7% of providers, were accredited to the NHIS by 31 December 2022.^29^ As shown in table 1, the three provinces with the most providers were Lusaka, Copperbelt and Southern, which had 53.9%, 18.9% and 10.6%, respectively. Lusaka and Southern Provinces were selected as the study sample as they had more private providers accredited to the NHIS in urban (Lusaka) and rural (Southern) provinces. The study included at least one private provider from hospitals, eye hospitals, hospices, clinics, pharmacies, laboratories, diagnostic centres and optician providers.

**Table 1:** Characteristics of accredited providers with the Zambia NHIS

Characteristics	Frequency	Percentage
Overall provider accreditation status		
Private (for-profit)	180	54.7%
Government	95	28.9%
Not-for-profit	53	16.1%
Mines facility	1	0.3%
Private providers location		
Central	6	3.3%
Lusaka	97	53.9%
Copperbelt	34	18.9%
Eastern	8	4.4%
Southern	19	10.6%
Western	3	1.7%
North Western	5	2.8%
Northern	5	2.8%
Muchinga	0	0.0%
Luapula	3	1.7%
Private provider location status		
Urban	131	72.8%
Rural	49	27.2%
Private provider type		
Hospital	14	7.8%
Eye hospital	3	1.7%
Hospice	3	1.7%
Clinic	27	15.0%
Dental clinic	10	5.6%
Diagnostic centre	8	4.4%
Laboratory	7	3.9%
Optician	55	30.6%
Pharmacy	53	29.4%

NHISNational Health Insurance Scheme

In total, we interviewed 30 private providers. Respondents were, in most cases, the facility manager. Almost all the interviewed facility managers were also providers with additional curated experiences for all providers within the facility. They were able to discuss the facility’s experiences with authority. Interviews lasted 32–71 min (see table 2 for additional interview characteristics). Participation in this study was voluntary. All respondents consented to take part in the study. During the interview, the respondents had the opportunity to refuse to answer questions they were not comfortable with and also had a choice to cancel the interview entirely. At all stages of recruitment, interview, data collection, analysis, dissemination and data storage, anonymity and confidentiality were maintained.

**Table 2:** Characteristic of the study sample, interview and accreditation period

Characteristics	Frequency	Percentage	Mean (±SD)
Study sample			
Number of private providers	180	100%	
Sample (Lusaka and Southern)	116	64%	
Number of interviews relative to the study population	30	16.7%	
Number of interviews relative to the study sample	30	25.9%	
Geographical status			
Urban (Lusaka province)	22	73.3%	
Rural (Southern province)	8	26.7%	
Provider-type interviewed			
Hospital	2	6.7%	
Eye hospital	2	6.7%	
Hospice	1	3.3%	
Clinic	6	20.0%	
Dental clinic	1	3.3%	
Optician	5	16.7%	
Pharmacy	9	30.0%	
Laboratory	3	10.0%	
Diagnostic	1	3.3%	
Interviewee sex			
Male	22	(73.3)	
Female	8	(26.7)	
Interview duration (in min)			
Total duration	1498		
Mean			49.9 (10.5)
Maximum	70.5		
Minimum	31.5		
Period since accreditation (in years)			
Total years since accreditation	60		
Mean			2 (0.83)
Maximum	3		
Minimum	1		

SDStandard Deviation

### Data collection and analysis

Based on the previous studies and stakeholder feedback, the study team developed a draft interview guide (online supplemental file 2). The interview domains explored provider perceptions before accreditation, their experiences during implementation and their recommendations. Additional probing questions followed the dynamics of each interview. One of the authors, WMS, conducted all the interviews.

All interviews were recorded on Zoom, transcribed verbatim and anonymised. Two authors, WMS and JS, read the transcripts several times to familiarise themselves with the data. We applied qualitative content analysis following Dahlgren *et al*.^30^ Qualitative content analysis focuses on analysing the differences and similarities between parts of the text, understanding the context of the participants and interpreting manifest and latent messages. WMS and JS inductively devised a coding book from the data and applied it across all transcripts using a chartering form developed with Microsoft Excel. The online supplemental file 3 presents exemplary quotes from the analysis. We grouped the codes into categories and subcategories for analysis. Any currency conversions in the study used an average exchange rate of US$1 =K20 (June 2023).

### Patient and public involvement

Patients and the public were not involved in this research’s design, conduct, reporting or dissemination.

## Results

Data were coded and organised into the following categories: motivations, accreditation, service provision, reimbursement and sustainability. The following sections present these in detail.

### Motivations

More generally, private providers were primarily motivated by the potential to maximise profits by gaining new clients from the scheme’s compulsory coverage across Zambia.

We had realised as a facility that very few people are affiliated with other insurance companies. People kept saying, 'Please be a part of NHIMA so we can access the service'. We realised that when we are not a part of NHIMA, we will have few people we can offer services, but if we join NHIMA, we will care for many people, even the vulnerable. (Dental Clinic)

Providers also contrasted the payment challenges by private insurance providers with positive experiences from private providers already accredited with the NHIS.

Those who were not accredited with NHIMA at that time, when they found out that we were accredited with NHIMA, they used to ask us, 'Is NHIMA paying you on time?' So, that was the misconception. NHIMA is as good as private insurance. (Optician)

Several providers saw the introduction of NHIS as a way of expanding access to some population subgroups that would, otherwise, not be able to access private provider services because of service affordability.

Some people in Zambia cannot register for (private) insurance because of the premium. So, these people cannot come to our hospital; private hospital services are expensive. With NHIMA on board, such people can have these services. (Eye Hospital)

Private providers were additionally motivated to join the scheme to complement public providers by providing better patient experiences, including shorter waiting times and human resources, health technology and stock availability.

This system cannot work without the private sector. NHIMA tends to many people. So, this means that drugs run out quickly, and public facilities cannot deal with that quick turnover of stock. (Dental Clinic)

### Accreditation

The NHIMA required providers seeking accreditation to demonstrate service readiness and availability following standards in the chosen service category.

NHIMA wants to make sure that you can provide services. NHIMA would inspect to make sure the facility has the capability. These other companies sign the contract even before they visit the facility. (Hospital)

In addition to the explicit accreditation criteria, private providers noticed that providers with unique services and those in rural areas were more likely to be accredited. Providers felt that this approach increased equity in service access to the rural population.

NHIMA coming on board would include everybody and help us expand our services, especially in inaccessible areas like rural areas. (Eye Hospital)

The private sector perceived the accreditation fees as affordable compared with the accreditation return on investment. The private sector also expressed satisfaction with the accreditation renewal process.

Because the hospital was already running, K12 000 was not a big deal to us. It is not a big deal anywhere. Looking at how many patients we have, we can pay that annually. It is a small amount. (Eye Hospital)

Some providers appreciated the responsiveness in communication during the entire process of accreditation and contracting. However, others felt a need to increase transparency in the accreditation process, including instituting an online mechanism to help them track the process.

Most of it, we are operating in dark shadows. When information is only known from one end, the other end suffers more because you do not know what follows or what is supposed to be done. You do not know if you will be accredited, if you need to improve, or if they are still reviewing your application. (Optician)

### Service provision

#### Scheme membership

The providers were concerned about the level of knowledge on enrolments, contributions and access to the benefits package with the general public and members of the NHIS.

When NHIMA was coming, we all thought it was a scam to get money out of people. So, there is a need to sensitise people on the benefits of being on NHIMA. (Clinic)

Providers considered the expected informal sector contribution of K60 (US$3) per month affordable. The buy-back option allowed members immediate access to the NHIS if they paid the entire 4-month contribution. Due to the seasonality of informal sector incomes, providers encouraged the initiation of phone reminders and bulk contributions.

NHIMA has many people using it because of the manageable monthly contribution. These other insurance companies, members are paying more. (Hospital)

#### Benefits package

Providers credited the Zambia NHIS for excluding services, such as teeth bleaching and sunglasses, and covering unique conditions, such as prostate and cervical cancer. However, under the hospital category, there were concerns that the 90-day limitation on inpatient stays did not account for hospice care. The drug package excluded drugs for abortion care, arthritis, low blood pressure, a range of sickle cell drugs and the go-to diabetic drugs, such as rosuvastatin and dapagliflozin. The eye package restricted members to a single check-up and one pair of glasses every 3 years. For the lab’s benefits package, providers noted the absence of blood tests for allergies, histology and cytology.

Looking at the number of patients we are receiving and the services being accessed, if it were not NHIMA, they would not manage. For those who are doing dialysis in a month, an entire session costs roughly over K50 000. So, if someone is using cash, it will be difficult. (Pharmacy)

All the providers included in the study were not involved in any process of designing or revising the benefits package.

From my experience, they have not yet revised (the benefits package). If they can give us a chance to write a very long list of the drugs that should be added, that could help us and the clients. We are the ones interacting with the clients daily. (Pharmacy)

#### Access to healthcare

Providers understood that preauthorisation is necessary as a gatekeeping measure to control misuse, but they generally perceived the process as unreasonably long.

Initially, there was no preauthorisation for a root canal. Somewhere, midway NHIMA pushed it to be under preauthorisation as well, which has yet to work well. You are forced to remove many teeth that you can save because NHIMA has delayed responding to give you the go-ahead. So, you are forced because the patient is in severe pain. (Dental Clinic)

Private providers identified additional bureaucratic procedures, including requiring patients to have a prescription bearing a claim number and stamp, a laboratory request form, a referral requisition form and codes authorising their use of the sought service. In addition to the required referral documentation, inpatients presented a doctor’s written consent indicating their incapacitation.

As an accredited pharmacy, we have been given specific instructions by NHIMA that you can only attend to a patient once they have all the correct documentation. If they do not, you have to send them back. On a human level, it feels terrible because you are sending a sick person back to the hospital. (Pharmacy)

Many of the providers interviewed believed that NHIMA staff in public hospitals could do more to guide enrolled members in determining what documentation is needed. Providers recommended digitalising the patient pathway to track services consumed and reduce physical documentation.

Most people sitting on the NHIMA desk in these hospitals are not qualified to attend to clients at that stage. You find that we, the private sector, have to teach the patient how they should go about the whole procedure. So, that is extra work for us and the patient’s finances in terms of transportation. (Pharmacy)

Several respondents expressed concern about NHIMA changing the restrictions and gatekeeping functions for services in the benefits package. For example, NHIMA changed the access protocol for optician providers to restrict patient eye testing to only public NHIS-accredited providers.

We get many inquiries like, do you accept NHIMA? I need to get glasses using them, and then we send them to the hospital. After that, only 10% of people will return, and 90% will say they do not have time to go to the hospital. There are long queues, and it takes time. This new protocol is troubling. (Optician)

### Service readiness and availability

All providers interviewed reported that NHIS members represented most of their clientele. A significant portion of these clients represented the informal sector and senior citizens. Many private providers reported overcrowding in their facilities. To mitigate overcrowding, in one instance, a provider was finalising the construction of a new hospital to cater entirely to NHIS members, separating them from cash and private insurance-paying clients.

The moment NHIMA came on board, we saw hospitals being full, and at our hospital, some people started to complain that they used to come into our hospital straight and be attended to, but they had to queue now. Because there are so many people coming on board. (Eye Hospital)

According to respondents, some private providers complement the public sector by offering all services under one roof to maximise service delivery and avoid referrals. Private providers perceived that providing more comprehensive services helped decongest public facilities.

We have improved our services by expanding, investing and reinvesting the generated revenue into growth, quality healthcare and stock. Almost everything is offered in-house. (Clinic)

Stockouts and drug shortages are common challenges in public health facilities in Zambia. To mitigate this, some private providers reported signing memorandums of understanding with suppliers to ensure that essential drugs and supplies are always available. Others built storage facilities to support their distribution.

It is business. You must be ready to meet your client’s demands in business. As a private sector, we always ensure that our drugs, medicine and surgical supplies are always available to meet our clients' demands. So, to have a shortage of that is quite rare. (Clinic)

### Reimbursements

#### Reimbursement tariffs

NHIMA offered predetermined and fixed provider tariffs for the duration of the accreditation with private providers. NHIMA applied specific diagnostic-related groups (DRGs) for hospitals and clinics for general illness consultation, laboratory and diagnostic investigations, treatment and medications. In addition, NHIMA used a fee-for-service tariff structure for some selected and expensive interventions.

The fee applies for consultation, registration, laboratory investigation, medicines and medical supplies issued and is limited to three visits. (Hospice)

In most cases, the hospital and clinic-based providers interviewed perceived reimbursement using the DRG as lower than the standard market prices for cash-paying and private insurance providers. Different from the fee-for-service, the DRG reimbursement, which grouped services for the treatment of a condition into a single tariff, was seen to disadvantage providers compared with when the services were reimbursed separately. Consequently, some providers referred patients outside their facilities to shift costs.

The tariff lump sum limits me in terms of what medication to give and what duration to give. So, most things are referred to a pharmacy when we can offer that service ourselves. Even in terms of laboratory services, my only option is to refer the patient to the laboratory to share the costs. (Hospital)

Pharmacy, laboratory and diagnostic providers generally regarded their fee-for-service tariffs as cost-reflective. Although in cases where their drug price was higher than the NHIS tariff, pharmacies were incentivised to use a cheaper generic drug, even when a known better alternative existed.

The prices are almost the same. Some drugs are less expensive compared with our prices. So, it is up to us to choose which costs are up to that price. We usually adjust by looking at the price, which is the system and our prices. (Pharmacy)

Overall, providers believed that the tariffs could be more responsive to market dynamics perpetuated by, among other things, the kwacha depreciation.

What people need to understand about the medical product industry in Zambia, or worldwide, is that prices change every week. So, if the tariffs stay the same, pharmacies are making more and more losses. (Pharmacy)

#### Claims process

All providers interviewed preferred online to paper-based claims. Providers used paper-based claims when inpatients could not be present, a member had duplicate accounts, senior citizens had non-readable fingerprints, patients' information was not migrated, online systems were down or client profiles had errors.

The online claim process is much better. It does not need you to do as much verification and paperwork. (Pharmacy)

The processing and feedback on paper-based claims were perceived to take longer than those submitted online. Some facilities made it a policy to avoid clients requiring paper-based claims.

What we have decided to do is not to work with paper claims. We provide the e-service to ensure that whatever is provided is accurate. (Clinic)

#### Claim rejections

Private providers reported experiencing high claim rejections, sometimes as high as 40%. Providers perceived paper-based claims as more likely to be rejected than online claims. Providers reported reasons for claims rejections as duplicate claims, failure to include the reference number on the prescription, uploading a prescription on a different client account, undertaking a service that did not receive authorisation and claims on the same patient for more than one diagnosis on the same day.

Most facilities were unaware of entering the claim reference number into the system and ensuring it was written on top of the prescription. So, it surprised us that we received rejections because we did not know. Even in the hospital, they did not know. Sometimes, they would just write it behind the prescriptions. (Pharmacy)

According to the providers interviewed, most rejections were due to NHIMA staff errors and the claims system’s inability to be automated. The high rate of rejections reportedly affected private providers' financial and service delivery performance.

When we send our people to their offices, they say it was not your fault but NHIMA’s. We do not expect faults from them. (Pharmacy)

#### Payments

Overall, private providers were happy with the NHIMA’s payment cycle. A few facilities reported longer payment durations beyond the contractually agreed-on period. While payments for paper-based claims took longer, providers acknowledged their businesses being cushioned by the consistent payments on online claims.

NHIMA ranks number one. It is unlikely that claims would go beyond 30 days. In a month, NHIMA sometimes makes up to three to four settlements, which is quite motivating and rewarding for a business. (Clinic)

On the other hand, all providers that submitted claim reconciliations on rejections had yet to receive payments despite following all the required procedures.

Right now, we are still fighting for the rejection. They have not paid for anything since NHIMA started. The most challenging issue when it comes to NHIMA is the rejections. (Pharmacy)

### Fund sustainability

Private providers reported experiences of fraud, moral hazard, adverse selection and provider-induced demand as threats to sustainability.

There were reported cases of colluding between providers and NHIS members with the intent of defrauding the scheme.

Many people (providers) were making false claims. They (NHIS members) were conniving with the service providers, and then they would make a claim and share. (Clinic)

The adverse selection was mainly with the informal sector, as while membership was mandatory in theory, it was voluntary in practice. Consequently, there was an incentive for the informal sector to only contribute to the scheme when using a service.

Some people would join NHIMA only to access the service. They come here, and when we tell them, it will cost them so much, they will say, 'Okay, I will join NHIMA'. (Hospital)

Providers observed high cases of moral hazards before introducing the eye care gatekeeping protocol and biometric fingerprint system.

At times, you will find a patient who saw the doctor in the morning and was not given treatment. But if they go home, they will still return in the evenings and require seeing a doctor with the same complaints. We have instances where somebody is coming in to see a gynaecologist, but they will still want to see a general doctor on the same day at the same time. (Hospital)

The cases of provider-induced demand included the use of NHIMA membership details for non-members at facilities of first contact. Although some second point-of-contact providers picked up on these experiences, there were no provisions for whistleblowing. The second point-of-contact providers argued that, since they only attended to clients from the first point-of-contact providers, there was only an incentive to induce demand if both providers colluded.

Most of us in the private sector are just here for the money. We charge people for things that they are not supposed to pay! It helps us to be regulated and to do the right thing. Sometimes, it is just mind games; we know that NHIMA has money, and they will pay us. Before they run out of money, we want it all. (Optician)

Although the NHIMA seemed to meet the current obligations, private providers were worried about the sustainability of the NHIS. They noted high service intake in the private sector by clients who previously would not have been able to access it, particularly, senior citizens who do not contribute to the fund.

I do not know their sustainability plan; that is my worry. Others argue that the 1% is enough, but I argue that the 1% is not enough. A few weeks ago, when NHIMA offices shut down, some said, 'Oh no, they are struggling now; they are shutting down'. Having some form of sustainable plan allows people to contribute to their vision. (Clinic)

As the population progressively joined the NHIS, providers recommended developing top-tier and add-on services to remedy the crowding out of private insurance companies.

Anybody can afford to contribute to NHIMA. Private insurance is only for high-class people. Private insurance companies should tie up with NHIMA… It would be like a third party, where the primary insurance company should be NHIMA. (Pharmacy)

While the NHIMA was under the Ministry of Labour and Social Services, providers called for continued involvement by the Ministry of Health, as health is the core function of the NHIS. Private providers were also concerned that their public provider counterparts had additional funding from the government and, thus, were less incentivised to be efficient in the delivery of NHIS services.

Whether NHIMA is there or not, public providers know that they will raise their revenue because they do not just depend on what the insurance (NHIMA) gives to the hospital. (Pharmacy)

## Discussion

The results show that private providers were motivated to join the NHIS to increase profits and complement public providers. They perceived the accreditation fees as affordable. However, private providers regarded the restrictions and exclusions of health services and the bureaucratic authorisation protocols as constraining access to the needed services by patients. More generally, the private providers acknowledged the presence of overcrowding and reduced service quality in some of their facilities. At the same time, they perceived their complementarity in improved service readiness and availability and member choices as a success. Providers generally perceived the reimbursement model as positive, although there were opportunities to shift costs to other providers. Resubmissions for rejected claims were not honoured across providers included in this study.

Our findings show that many initial fears among private providers appear to have been curbed. In research conducted at the onset of the Zambia NHIS implementation, stakeholders perceived the benefit of including the private sector as an opportunity to integrate them into the health system, decongest public facilities, increase member choices and complement public providers in service readiness and availability. However, there were concerns about possible accreditation, claims and payment delays and challenges with the referral system and equity in service delivery.^21 23^

Like Ghana and Kenya, where the private sector was seen as self-seeking, this study also identified profits as a primary motivation.^31^ Furthermore, the private providers seeking to access large client volumes and mitigate crowding out of cash and insurance-paying clients have also been observed elsewhere.^32^ As in this study, the private sector in other countries complemented the public sector through service readiness and availability.^31 32^ In the Zambia NHIS, all private providers, including hospitals, clinics, opticians, pharmacies, laboratories and diagnostics, apply for accreditation consideration, a system practised in other jurisdictions.^33^ However, unlike the Zambia case, some countries only accredit large service providers as hospitals.^13 34^ To be contracted, providers in Zambia and other countries such as India and Thailand must meet accreditation requirements, including the availability of medical personnel such as doctors.^32 34^ Unlike Zambia, where accreditation contracts are signed independently by each provider, contracts in other countries allow providers to subcontract other facilities to provide insured services.^34^ Challenges to extending the compulsory nature of the NHIS are broader than in Zambia alone; in Nigeria, for example, while the scheme is mandatory, informal sector inclusion has remained significantly voluntary.^33^

As seen elsewhere, providers in Zambia identified the informal sector as using the scheme’s services the most.^35^ However, other countries are exploring expanding service provision to additional underserved populations to meet the service gaps in pursuing UHC.^36^ In this study, preauthorisation was a significant challenge to accessing health services when needed. Other countries have digitised the preauthorisation process and included escalation protocols to ensure timely preauthorisations.^32^ In addition, the Zambia NHIS had a functional referral system, as observed in other countries implementing the NHIS.^31^

The Zambia NHIS provider payment mechanism uses the DRG, fee-for-service and flat rate tariff structure. Other countries, such as Thailand, also use a retrospective DRG for inpatient, although under a global budget and a fee-for-service to interventions that would financially threaten providers; however, in contrast, it applies prospective capitation for outpatient services.^34^ Other countries, such as Ghana, have continued exploring appropriate payment mechanisms to reduce fraud, such as adopting the capitation approach.^36^ Claims rejections were as high as 40% for some providers in this study compared with countries, such as India, with an estimated lower rejection rate of 5%.^32^ The private-sector’s call for fund sustainability evidence to be transparently made available to all stakeholders is also supported in the literature.^35 37^ The study evidence has shown that provider and consumer behaviours are potential threats to the sustainability of the NHIS. This concern has been expressed by the Government of Zambia and relevant stakeholders, who have alleged that the private sector exploits the scheme with fraudulent claims to maximise profit.^38^,^40^ As in other jurisdictions, the NHIMA has carried out initiatives to support the sustainability of the NHIS through contracting and gatekeeping measures.^34^ In June 2024, NHIMA revoked the accreditation of 13 private health providers due to violations of contractual obligations, including fraud.^38^

After this study data collection was finalised, three significant developments in the implementation of the NHIS could affect the interpretation of our results. First, the NHIMA removed the buy-back option, which allowed members to consume services immediately after paying their 4-month contributions by 1 January 2024.^39^ According to the Minister of Labour and Social Services, the government removed the buy-back option as the NHIS month-on-month claims exceeded the revenues and threatened the fund’s solvency.^39^ The performance of this policy change concerning seasonality in informal sector incomes and challenges in maintaining monthly premiums will need to be explored. Second, in October 2023, the regulatory governance of the NHIS was moved from the Ministry of Labour and Social Services to the Ministry of Health barely 2 years after its switch between the two ministries.^40^ There is a need for additional research that will review the NHIS governance policy and its impact on UHC goals. Third, in May 2024, the NHIMA revised the benefits package, removing access to spectacles, among other benefits, and introduced copayments on certain services in a move to curtail unsustainable expenditures alleged to have been perpetuated by the private sector. This decision was rescinded almost immediately after stakeholders appealed for a more inclusive and consultative revision process.^41^

As a limitation, this study did not extend the qualitative analysis with document reviews or obtain additional insights from stakeholders other than the private sector. Future research should broaden the scope of perspectives by including further evidence from the public sector. While the study attempted to make a nuanced analysis across providers, the limited number of included providers, namely hospitals, made this challenging. Future research could explore providers' experiences within the same service provision category. While this study has identified the complementary roles of the private and public sectors, illustrating an effective referral mechanism and highlighting growing concerns about service quality caused by overcrowding, future research should delve deeper into complementary arrangements to produce insights that enhance complementarity. In addition, future studies should explore administrative data from the NHIS on enrolments, accreditation, service utilisation, claims, rejection and payments to ascertain the fund’s performance against UHC objectives.^42^

## Conclusion

This study explored private provider’s experiences with implementing NHIS in Zambia. Private providers' initial concerns about potential high accreditation fees and accreditation, claims and reimbursement delays subsided. They perceived their participation as improving equity in service access by the rural population and those financially excluded, complementing public facilities with service readiness, availability and member choice. Although some challenges remain, our study has shown an overall positive experience among private-sector providers with the NHIS implementation.

## supplementary material

10.1136/bmjopen-2024-092047 online supplemental file 1

10.1136/bmjopen-2024-092047 online supplemental file 2

10.1136/bmjopen-2024-092047 online supplemental file 3

## Data Availability

All data relevant to the study are included in the article or uploaded as supplementary information.

## References

[1] World Health Organization (2010). The world health report: health systems financing: the path to universal coverage.

[2] World Health Organization and International Bank for Reconstruction and Development / The World Bank (2023). Tracking universal health coverage: 2023 global monitoring report.

[3] Kodali PB (2023). Achieving Universal Health Coverage in Low- and Middle-Income Countries: Challenges for Policy Post-Pandemic and Beyond. Risk Manag Healthc Policy.

[4] Lagomarsino G, Garabrant A, Adyas A (2012). Moving towards universal health coverage: health insurance reforms in nine developing countries in Africa and Asia. Lancet.

[5] Fenny AP, Yates R, Thompson R (2021). Strategies for financing social health insurance schemes for providing universal health care: a comparative analysis of five countries. Glob Health Action.

[6] Cashin C, Dossou J-P (2021). Can National Health Insurance Pave the Way to Universal Health Coverage in Sub-Saharan Africa?. Health Systems & Reform.

[7] Spaan E, Mathijssen J, Tromp N (2012). The impact of health insurance in Africa and Asia: a systematic review. *Bull World Health Organ*.

[8] Erlangga D, Suhrcke M, Ali S (2019). The impact of public health insurance on health care utilisation, financial protection and health status in low- and middle-income countries: A systematic review. PLoS One.

[9] Mathauer I, Dale E, Jowel M (2019). Purchasing of health services for universal health coverage: how to make it more strategic? (WHO/UHC/HGF/PolicyBrief/19.6). Licence: CC BY-NC- SA 3.0 IGO.

[10] Sakamoto H, Aya I, Tangcharoensathien V (2023). The role of the private sector: challenges and opportunities in Asia for achieving universal health coverage.

[11] Montagu D, Goodman C (2016). Prohibit, constrain, encourage, or purchase: how should we engage with the private health-care sector?. Lancet.

[12] Dalinjong PA, Laar AS (2012). The national health insurance scheme: perceptions and experiences of health care providers and clients in two districts of Ghana. Health Econ Rev.

[13] Suchman L (2018). Accrediting private providers with National Health Insurance to better serve low-income populations in Kenya and Ghana: a qualitative study. Int J Equity Health.

[14] Odeyemi IAO (2014). Community-based health insurance programmes and the National Health Insurance Scheme of Nigeria: challenges to uptake and integration. Int J Equity Health.

[15] Mohammed S, Bermejo JL, Souares A (2013). Assessing responsiveness of health care services within a health insurance scheme in Nigeria: users’ perspectives. BMC Health Serv Res.

[16] Umar S, Fusheini A, Ayanore MA (2020). The shared experiences of insured members and the uninsured in health care access and utilization under Ghana’s national health insurance scheme: Evidence from the Hohoe Municipality. PLoS One.

[17] Kwon S (2009). Thirty years of national health insurance in South Korea: lessons for achieving universal health care coverage. Health Policy Plan.

[18] Siddiqi S, Aftab W, Venkat Raman A (2023). The role of the private sector in delivering essential packages of health services: lessons from country experiences. BMJ Glob Health.

[19] Agyepong IA, Abankwah DNY, Abroso A (2016). The “Universal” in UHC and Ghana’s National Health Insurance Scheme: policy and implementation challenges and dilemmas of a lower middle income country. BMC Health Serv Res.

[20] Government of Zambia (2018). The National Health Insurance Act [no.2 of 2018].

[21] Sinjela KM, Simangolwa WMW, Hehman L (2022). Exploring for-profit healthcare providers’ perceptions of inclusion in the Zambia National Health Insurance Scheme: A qualitative content analysis. PLoS One.

[22] Government of Zambia (2019). Statutory Instrument No. 63 of 2019.

[23] Osei Afriyie D, Masiye F, Tediosi F (2023). Purchasing for high-quality care using National Health Insurance: evidence from Zambia. Health Policy Plan.

[24] O’Brien BC, Harris IB, Beckman TJ (2014). Standards for reporting qualitative research: a synthesis of recommendations. Acad Med.

[25] Ministry of Health (2022). 2022-2026 National health strategic plan.

[26] Ministry of Health (2017). Health financing strategy: 2017 – 2027 towards universal health coverage for Zambia.

[27] World Health Organisation (2023). Global Health Expenditure Database.

[28] Ministry of Health (2017). National health strategic plan 2017-2021.

[29] National Health Insurance Management Authority (2022). NHIMA List of Accredited Healthcare Facilities 22/06/2022.

[30] Dahlgren L, Emmelin M, Hällgren Graneheim U (2019). Qualitative methodology for international public health, 3rd edn.

[31] Suchman L, Hart E, Montagu D (2018). Public-private partnerships in practice: collaborating to improve health finance policy in Ghana and Kenya. Health Policy Plan.

[32] Furtado KM, Raza A, Mathur D (2022). The trust and insurance models of healthcare purchasing in the Ayushman Bharat Pradhan Mantri Jan Arogya Yojana in India: early findings from case studies of two states. BMC Health Serv Res.

[33] Alawode GO, Adewole DA (2021). Assessment of the design and implementation challenges of the National Health Insurance Scheme in Nigeria: a qualitative study among sub-national level actors, healthcare and insurance providers. BMC Public Health.

[34] Marshall AI, Witthayapipopsakul W, Chotchoungchatchai S (2023). Contracting the private health sector in Thailand’s Universal Health Coverage. *PLOS Glob Public Health*.

[35] Witter S, Garshong B (2009). Something old or something new? Social health insurance in Ghana. BMC Int Health Hum Rights.

[36] Aikins M, Tabong PT-N, Salari P (2021). Positioning the National Health Insurance for financial sustainability and Universal Health Coverage in Ghana: A qualitative study among key stakeholders. PLoS One.

[37] Simangolwa WM, Mbonigaba J, Govender K (2024). Health technology assessment for sexual reproductive health and rights benefits package design in sub-Saharan Africa: A scoping review of evidence-informed deliberative processes. PLoS One.

[38] News Diggers Zambia (2024). 13 health care providers lose NHIMA accreditation.

[39] News Diggers Zambia (2024). Buy back option threatened NHIMA’s existence – Tambatamba.

[40] News Diggers Zambia (2024). Return of NHIMA to MoH will bolster universal health coverage – Moyo.

[41] News Diggers Zambia (2024). NHIMA U-Turns On Spectacles, Dental Benefits.

[42] Simangolwa WM, Govender K, Mbonigaba J (2024). Health technology assessment to support health benefits package design: a systematic review of economic evaluation evidence in Zambia. BMC Health Serv Res.

