# Supplementary material for: Role of private providers in the implementation of the national health insurance scheme in Zambia: a qualitative study of perceptions and experiences
Source: BMJ Open. 2025 Feb 10;15(2):e092047. doi: 10.1136/bmjopen-2024-092047 (PMC11831299; doi:10.1136/bmjopen-2024-092047)
Supplement: online supplemental file 2 [file bmjopen-15-2-s002.docx]

**Supplementary File 2: Interview Guide**

1. What was your motivation for joining the NHIS?

- Probe on primary benefits accrued to provider: increased business portfolio, service quality and efficiency improvements, client volumes, and profits.
- Probe on secondary benefits: organisational reputation, new markets on insurance payers and UHC (population coverage, services, and financial barriers)

1. To what extent have your expectations in question 1 above been met? Probe.
2. In your experience, what are the steps involved in the accreditation process?

- Probe on the process, clarity in forms, fees, duration, communication and feedback, complaints responsiveness, and renewals
- What factors influence the award of accreditation? Probe the age of the organisation, geography, and service differentiation.

1. Were the experiences in question 3 above different from your perceptions of the accreditation process? Probe.
2. How does a client access your services, and to what extent do your services, as a private provider, complement the provision of care to NHIS members?

- Probe on health services, standards, quality, availability of health technology, human resources, altitudes, and geographical distribution
- Probe on services and product demand, availability, and stockouts

1. Are any of your experiences to question five above different from your initial perception before joining the NHIS? Have they evolved since initiation? Probe.
2. What steps are involved in making claims to the NHIS for your category of service provision?

- Probe the process, claims vetting, duration, information system, and protocol for claims complaints.

1. How does the NHIS claims process compare to private health insurance providers? Have they evolved since the initiation of the NHIS? Probe
2. Describe the provider payment mechanism used for your category of service provision.

- Probe on the fee for service, advance payment, diagnostic-related grouping
- Probe on tariffs structure: segmented into components (consultation fees, disease management) or grouped for service type.
- Probe on cost reflectiveness of tariff structure

1. How does the provider payment mechanism for the NHIS described in question 9 above compare to that provided by private insurance providers? Probe on perceived changes and adapted roles of private insurance providers in the face of NHIMA
2. How is the private sector organised to support the better implementation of the NHIS?

- Probe formal or informal group meetings and dialogue with NHIS

1. Do you want to add anything about your perceptions/experiences of NHIS? Is there something important that I have missed?
