# Supplementary material for: Role of private providers in the implementation of the national health insurance scheme in Zambia: a qualitative study of perceptions and experiences
Source: BMJ Open. 2025 Feb 10;15(2):e092047. doi: 10.1136/bmjopen-2024-092047 (PMC11831299; doi:10.1136/bmjopen-2024-092047)
Supplement: online supplemental file 3 [file bmjopen-15-2-s003.docx]

**Supplementary File 3: Exemplary Quotes from the Analysis**

| **Provider Type** | **Category** | **Sub-Category** | **Code** | **Code Summary** | **Quotes** |
| --- | --- | --- | --- | --- | --- |
| Pharmacy | Motivation | Business Opportunity | Profit | (A) The primary motivation is to increase sales and thus profits in addition to secondary motivations. (B) the introduction of the NHIS reduced the volume of clients that would buy medications in cash. As a consequence, the facility was compelled to get accredited to retain the new form of clients now with NHIS. | (A) "That could help us business wise, and we can also be of help to the society at large." (B) "Clients that would usually just come to buy the medication reduced. So, we thought, maybe if we got accredited to NHIMA the number of clients will actually increase" |
| Eye Hospital | Motivation | Business Opportunity | Profit | (A) The NHIS promised to be countrywide, include everyone, and as such, be a big market opportunity for the provider. | (A) "NHIMA being a national wide insurance, the clientele base is big" |
| Optician | Motivation | Universal Health Coverage | Services | (A) Able to provide quality services in a short amount of time. This is important for productivity – a client has places to be and work to do. Government facilities make them wait longer than they should | (A) " Nowadays people are busy, they need to work, they need to do other things. You put me to wait for consultation only for 3 hours or 2 hours at the government institution, but you come to a private sector it's only take 5 minutes within that you’re done" |
| Dental Clinic | Accreditation | Accreditation Process | Process and Factors | (A) The initial process is tedious whilst accreditation is easy, if standards are maintained. (B) The initial accreditation process seems longer, perhaps because a facility is waiting on action by NHIMA. Otherwise perceived to be slightly over average in duration (C) All the processes and documents were clear. (D) The accreditation process follows clear requirements on the standards that the private sector must put in place so as to be accredited. | (D) "They are a lot of things that you expected to put in place for you to meet the standards to be accredited. There is a certain standard especially to a private facility, that you need to meet for NHIMA to be sure that you are able to deliver quality work" |
| Clinic | Accreditation | Accreditation Process | Additional Factors | (A) HPCZ: Accreditation was easy as the (1) has always met the HPCZ licensing standard, (2) NHIMA follows the HPCZ standards | (A) "It was not a challenging process because we were ready. We are assessed by the health profession council of Zambia to meet the requirement which allows a health facility to offer good quality health care to the patient" |
| Hospital | Accreditation | Communication and Feedback | Communication and SOPs | (A) Facility physical training was 1 day. The training involved claims processes. However, training was not enough as the facility faced a lot of challenges in implementation and had to get additional support from NHIMA. (B) Changes to the protocol are necessary as NHIMA grows. But stakeholders should be consulted and informed so as to be ready | (B) "NHIMA is growing and most of the changes are necessary to improve service provision on their own part. But providers also they need to be consulted at least on time so that they are ready too" |
| Clinic | Service Provision | Service Availability and Readiness | Demand | (A) the demand for facility services has doubled. The facility welcomes this additional demand. As a mitigation on stockouts, the facility has a 1-month advance stock capacity. (B) Facility Preference: There is a preference for private facilities over government facilities due to the friendliness of the staff | (A) "We didn't expect that we would be getting what we are getting and how people are coming…. Clients are coming in every day. We are busy! (B) "That one is hundred percent, we’ve got very good reception. We are very welcoming" |
| Pharmacy | Service Provision | Service Availability and Readiness | Referrals | (!) Most of the NHIMA Desk Attendants are not qualified. They are not helpful to the patient in ensuring the patient has (1) a Stamp, (2) an Authorisation code for drugs that need it (3) that the prescription has been checked for drug API. Their inefficiency costs patient’s out-of-pocket payments | (A) “Clients don't even know the price. They spend more transport coming to our facilities. If they can be told to say this medication maybe cost this much and this much, at least they have a clear picture to say I can't spend transport on something that I can just buy" |
| Clinic | Service Provision | Admin | Membership | (A) Members can receive services almost immediately. Use NRC to verify the activeness status (B) None Paying members: (1) In case of the employer not remitting, the employer is required to write a letter indicating they will continue to pay for the employee and with 72 hrs, authorisation can be granted (2) case of informal sector, the required to pay the entire balance to receive care. (C) (1) NHIMA is affordable for the services offered | (C) "Nobody plans to get sick, when one is sick, you find that they don't have liquidity in terms of cash, but NHIMA will always come on board and cover them. The percentage, one should be able to subscribe is entirely depending on how much they earn or how much they make which is almost affordable for everyone" |
| Optician | Reimbursement | Payments | Payments | (A) Used to be okay but has gone down. They are not making payments according to the contract that was signed. Their feedback is that they have a lot of facilities to pay for. (B) NHIMA has been meeting financial its obligations as specified in the SOP. This has earned the trust of the facility to continue to do business. This comes from a background of cadres, the government failing to minor its obligations | (B) "We at first were doubting them, but they started meeting the financial obligations, our relationship developed" |
| Clinic | Reimbursement | Tariffs and Fees | Tariffs and Fees | (A) Tariffs should be reviewed annually to align with global economic conditions (B) Tariffs are billed as a bundle under optician services and general consultation, which have specific bundled prices | (A) "it being a national scheme, it should be reviewed annually, so that it can align with what is prevailing in the global economy. Because, for the past 3, 4 years, they have put the same threshold for example under specs and then prices have increased" |
| Pharmacy | Reimbursement | Claims | Rejected Claims | (A) The rejection rate is high at 40% and impacts business profitability. (B) Rejection codes: There are rejections which do not have codes, and reconciliation on such is tedious as a letter has to be written to the DG which guarantees that the process will take a very long time. (C) There is less focus on processing rejections | (A) " When there are rejections, those rejections come with codes and then that code is aligned with a particular reason for the rejection. They are certain codes don't actually have any reason. So, you find that you contact NHIMA, they are saying, no, just write letter to the DG. What we do know is that the DG is a very busy person, so he's not going to really see from all this" |
| Eye Hospital | Sustainability | Fund Sustainability | Consumer and Provider Behaviours | A) Moral hazard and provider-induced demand: Possible. The facility maintains the need to test clients first and recommend alternative courses of action, including medical routes, rather than immediately using corrective lenses. To avoid abuse and misuse of resources, people in Zambia attach a certain level of nuttiness to wearing glasses. | (A) "People like it when they wear glasses, there’s this thing that is attached to glasses. But our hospital will say look we don't just give glasses, we need to test you, scan you to see whether you need the medication. It can easily be abused" |
| Pharmacy | Sustainability | Fund Sustainability | Fund Sustainability | (A) Adverse selection: Experience of Instances of patients that join NHIMA only because they have a medical problem. | (A) "Most of them join when there is a problem. So, they join today and they want to collect drugs today" |
| Pharmacy | Sustainability | NHIS Performance | NHIS Performance | (A) Leave no one behind: NHIMA has failed to bring everyone on board, especially the informal sector. (B) NHIMA should explore leveraging technology to accredit mobile providers (providers with a nurse onsite and a doctor elsewhere). | (A) "NHIMA has the 'leaving no one behind' statement. if they are trying to really achieve' the leaving no one behind', then there is need to ensure that people are informed about NHIMA services. They just need for a proper strategy. There isn’t that deliberate move to market the NHIMA service. The level of literacy in Zambia, is very low, especially in these underserved areas" |
