# Supplementary material for: Role of private providers in the implementation of the national health insurance scheme in Zambia: a qualitative study of perceptions and experiences
Source: BMJ Open. 2025 Feb 10;15(2):e092047. doi: 10.1136/bmjopen-2024-092047 (PMC11831299; doi:10.1136/bmjopen-2024-092047)
Supplement: Supplementary file 4 [file bmjopen-2024-092047.reviewer_comments.pdf]

PEER REVIEW HISTORY

BMJ Open publishes all reviews undertaken for accepted manuscripts. Reviewers are asked to complete a checklist review form and are provided with free text boxes to elaborate on their assessment. These free text comments are reproduced below.

ARTICLE DETAILS

Title (Provisional)

The Role of Private Providers in the Implementation of the National Health Insurance Scheme in Zambia: A Qualitative Study of Perceptions and Experiences

Authors

Simangolwa, Warren Mukelabai; Sundewall, Jesper

VERSION 1 - REVIEW

**Reviewer**	**1**
**Name**	**Katz , Aaron**
**Affiliation**	**University of Washington , Department of Health Services**
**Date**	**13-Sep-2024**
**COI**	**None**

The study objective is alluded to but never explicitly stated (i.e., "to understand private provider experiences with ..."

Concerning the appropriateness of the study design, I guess my question is whether talking mostly with clinic managers, as opposed to service providers themselves, is sufficient to understand "private provider's experiences." I don't know the Zambia context, so the answer may well be "yes."

In Table 2, the total number of interviews (30) is shown to be a 16.7% study sample; but given the sample provinces include 116 providers, isn't the correct number 25.8%?

The study and its results certainly provide one part of the picture of private provider experiences, but without also knowing public provider views on the same set of questions, isn't it difficult to understand those private provider views?

A few more detailed comments:

* Under "Reimbursements" p. 11, "Hospital and clinic-based private providers perceived some services within each DRG as too different ..." I'm not sure what this means, could you explain it a bit more clearly?

* Under Fund Sustainability p. 13, what is described as "moral hazard" is not that, in my view, but simply fraud (alluded to in the Discussion). Also, the quote on the page starting on line 53 is quite remarkable; I don't think this profit-seeking motivation gets enough play in the abstract or conclusion. This might be a point in which clinic managers and service providers differ?

Reviewer 2

Name Ssennyonjo, Aloysius

Affiliation Makerere University School of Public Health, Health Policy Planning and Management

Date 01-Oct-2024

COI None

---

bmjopen-2024-092047: The Role of Private Providers in the Implementation of the National Health Insurance Scheme in Zambia: A Qualitative Study of Perceptions and Experiences

First Review 1

General comment:

This is a well written piece of work in easily accessible language. It touches a very important topic on NHIS implementation in African settings especially within the private sector. The comments below are intended to enrich the work further and extend its policy and practical relevance with Zambia and beyond.

Specific comments:

A. Introduction
   1. Page 3, line 28: what were the changes in Ghana's provider payment systems.
   2. Page 3, lines 40-41: Why are Ethiopia and Pakistan struggling to incorporate the private sector?
   3. Page 3, line 42, do you mean NHIS members?

B. Methods
   1. Page 4 Lines 30-40:Some content on study setting/ context is erroneously covered under study participants. Revise the headings accordingly.
   2. Some background on the Zambian health system might add context to the study. For example, what is the proportion of public, PNFPs and Private providers across the entire health system? What is the population coverage of NHIS across the country in terms of enrollment? What are the other health financing mechanisms and what is the contribution of NHIS to the

total health expenditure? What are the current out of pocket expenditures by latest National Health accounts or other databases?

3. It is good to see the clarifications for including Lusaka and Southern provinces based on the urban- rural distinction. However, it is not apparent in the findings that these distinctions and comparisons were carried forward during analysis. Could the authors try to reflect on these contextual realities in their analysis? It would be useful to maintain a comparative perspective during the analysis to capture any similarities and differences in provider experiences between rural and urban settings?
4. Page 5 line 47: Were there other considerations for respondents' inclusion or exclusion beyond being the manager to ensure that the respondents had the required experiences in NHIS implementation? For example, looking into the duration of manager in this position at the selected facilities or overall management experience in private health sector in Zambia even if from another facility?
5. The heading "Data collection methods and interview guide" could be tweaked to include analysis

C. Results

1. General comment: The current findings are well written and easy to follow. The quotes affirm the insights in the preceding context. However, the current analysis could be deepened to enrich the study further.
   i. The quotes are used just to confirm the content alluded to in the text. It is not clear if there were no conflicting perspectives or nuances that could be reflected in the study. Each point has one quote as illustrative but it would be useful to look into divergences in quotes as well.
   ii. In some sections, there is an overreliance on insights from providers of special services such as eye and dental care. How confidently can these experiences extend to hospital and clinics that provide general services. A deeper focus on these distinctions among provider types would derive more nuanced findings. For example, how do the motivations of these special providers compare with those of hospitals and clinics that provide a broader service package?
   iii. The focus on rural and urban settings in choosing the same southern and Lusaka provinces is lost in the findings? What could be the reasons? Otherwise, it would be helpful to appreciate if these provider experience carry any rural-urban distinctions.

D. Discussion

1. The discussion does a good job to compare Zambia's experiences with those of other countries. It would be useful to refer to the key lessons and implications of these findings for policy and practice in Zambia and other countries implementing or considering NHIS.

i. There are usually concerns about NHIS being a 'trojan horse' towards privatization of health service delivery l there by undermining UHC goals. Private sector inclusion in health service provision is related to some unintended outcomes e.g crowding out public providers due to dysfunctional competition between public and private providers in same settings. Are the private providers in Zambia's NHIS indeed complimentary as suggested? If so, what makes it work? What could be learned from the institutional arrangements in place?
ii. How about the concerns about market failures such as moral hazard- specifically supplier-induced demand? What do these mean for the NHIS in Zambia or elsewhere
iii. The authors could also reflect on the data systems and claims verification processes in private sector: how easy is it to harmonize data systems and what safeguards exist or could be adopted to enrich data quality and curb fraud?

VERSION 1 - AUTHOR RESPONSE

Authors Feedback to Reviewer

SN	Comment	Feedback
**Review Comments to the Author**		
**Reviewer #1**		
1	The study objective is alluded to but never explicitly stated (i.e., "to understand private provider experiences with ..."	We thank the reviewer for their observation. Lines 189, has been adjusted to explicitly itemise the purpose of the study as advised.
2	Concerning the appropriateness of the study design, I guess my question is whether talking mostly with clinic managers, as opposed to service providers themselves, is sufficient to understand "private provider's experiences." I don't know the Zambia context, so the answer may well be "yes."	Thank you for highlighting feedback on the study design. Yes – Private provider facility managers in Zambia are often owners of the practice and services providers themselves. In addition, managers are better placed to curate the challenges and experiences across other practitioners within the facility, than a single provider within a section. We have adjusted the methods to include this explanation to enhance clarity of the design (see lines 252 and 254).
3	In Table 2, the total number of interviews (30) is shown to be a 16.7% study sample; but given the sample provinces include 116 providers, isn't the correct number 25.8%?	We thank the reviewer for identifying the erroneous allocation. Yes, the number of interviews represent 25.9% of the study sample. The 16.7% was in relation to the study population of 180. We have adjusted Table 2 to include a new row, comparing the number of

		interviews to the population and the sample, to enhance clarity.
4	The study and its results certainly provide one part of the picture of private provider experiences, but without also knowing public provider views on the same set of questions, isn't it difficult to understand those private provider views?	We agree with the reviewer and thank them for highlighting the evidence gap. As this study is a follow-up to a previous study that focused on the private sector inclusion in the NHIS, it was not within the jurisdiction of the objectives of the study to use a similar lens with the public providers. However, we acknowledged the value of such evidence in our limitation and have further included in lines 725 and 727 on this evidence gap and implications for future research.
5	A few more detailed comments: *Under "Reimbursements" p. 11, "Hospital and clinic-based private providers perceived some services within each DRG as too different ..." I'm not sure what this means, could you explain it a bit more clearly?	Thank you. For clarity as advised, we have deleted the sentence and replaced it with "Different from the fee-for-service, the DRG reimbursement, which grouped services for the treatment of a condition into a single tariff, was seen to disadvantage providers compared to when the services were reimbursed separately", in lines 494 to 496. Prior to this, the services grouped in the DRG was highlighted in lines 484 to 486.
6	* Under Fund Sustainability p. 13, what is described as "moral hazard" is not that, in my view, but simply fraud (alluded to in the Discussion).	Indeed so. We agree. We have introduced in the sentence in lines 583 and 584 to introduce in the 'fraud' component and maintained the quote to this reference. We have introduced a new quote in lines 602 to 606 to retain the issue of moral hazards.
7	Also, the quote on the page starting on line 53 is quite remarkable; I don't think this profit-seeking motivation gets enough play in the abstract or conclusion. This might be a point in which clinic managers and service providers differ?	Thank you for highlighting this important item. We have adjusted the abstract to include "… including inducing demand to defraud the fund" in line 39, to add to the sentence on cost shifting by the private sector.
	**Reviewer #2**	
1	This is a well written piece of work in easily accessible language. It touches a very important topic on NHIS implementation in African settings especially within the private sector. The comments below are intended to enrich the work further and extend its policy and practical relevance with Zambia and beyond.	We thank and appreciate the reviewer for the thoughtful feedback.

2	A. Introduction<br>1. Page 3, line 28: what were the changes in Ghana's provider payment systems.	Thank you. In lines 152 and 153 we have added additional context to the changes to Ghana's provider mechanism.
3	Page 3, lines 40-41: Why are Ethiopia and Pakistan struggling to incorporate the private sector?	We thank the reviewer for the clarifying request. Lines 165 and 166 have been included to provide clarity on Ethiopia and Pakistan, as advised.
4	3. Page 3, line 42, do you mean NHIS members?	Thank you. The sentence was directed private healthcare providers for the NHIS. We have added "with public providers" in line 175 after complementarity, to enhance clarity.
5	B. Methods<br>1. Page 4 Lines 30-40: Some content on study setting/ context is erroneously covered under study participants. Revise the headings accordingly.	We thank the reviewer in identifying the error of allocation of text. A new sub-section; study content has been introduced in line 203 to 224 and some sentences pulled from the section participants, as advised.
6	Some background on the Zambian health system might add context to the study. For example, what is the proportion of public, PNFPs and Private providers across the entire health system? What is the population coverage of NHIS across the country in terms of enrollment? What are the other health financing mechanisms and what is the contribution of NHIS to the total health expenditure? What are the current out of pocket expenditures by latest National Health accounts or other databases?	We appreciate the reviewer for highlighting this need. Lines 203 to 224 have been introduced to provide a summary of Zambia's health system, NHIS coverage, contributions of the NHIS to CHE and OOP estimates, as advised.
7	It is good to see the clarifications for including Lusaka and Southern provinces based on the urban- rural distinction. However, it is not apparent in the findings that these distinctions and comparisons were carried forward during analysis. Could the authors try to reflect on these contextual realities in their analysis? It would be useful to maintain a comparative perspective during the analysis to capture any similarities and differences in provider experiences between rural and urban settings?	Thank you. We compared the rural and urban areas for each theme and where differences were found, we highlighted these. For example, in lines 342 to 243. We also added this to the conclusion.
8	4. Page 5 line 47: Were there other considerations for respondents' inclusion or exclusion beyond being the manager to ensure that the respondents had the required experiences in NHIS implementation? For example, looking into the	Thank you. Yes – the facility managers were often providers with substantial experience in the facility and could speak authoritatively on the experiences of other providers with the facility. We have added a sentence to lines 253 to 255 to emphasise this.

	duration of manager in this position at the selected facilities or overall management experience in private health sector in Zambia even if from another facility?	
9	5. The heading “Data collection methods and interview guide” could be tweaked to include analysis	We thank the reviewer for this empowering suggestion. We have adjusted the section to read “data collection and analysis” in line 263.
10	C. Results<br>1. General comment: The current findings are well written and easy to follow. The quotes affirm the insights in the preceding context. However, the current analysis could be deepened to enrich the study further.	We appreciate this feedback and have attempted to incorporate the suggestions as advised throughout the manuscript to enrich the study.
11	The quotes are used just to confirm the content alluded to in the text. It is not clear if there were no conflicting perspectives or nuances that could be reflected in the study. Each point has one quote as illustrative but it would be useful to look into divergences in quotes as well.	We thank the reviwer and appreciate their insights on an effective analysis approach. We introduced divergent views as separate quotes within each section, where it was possible.
12	ii. In some sections, there is an overreliance on insights from providers of special services such as eye and dental care. How confidently can these experiences extend to hospital and clinics that provide general services. A deeper focus on these distinctions among provider types would derive more nuanced findings. For example, how do the motivations of these special providers compare with those of hospitals and clinics that provide a broader service package?	We appreciate the reviewer for this nuanced reflection and agree that this approach would ideally benefit the study. However, the differences in services delivered by the private providers made comparisons challenging. As the number of included hospitals was few, such comparisons would be spread too thinly for a broader interpretation. Following the reviewer observation, we have included this as a limitation on lines 728 to 729.
11	The focus on rural and urban settings in choosing the same southern and Lusaka provinces is lost in the findings? What could be the reasons? Otherwise, it would be helpful to appreciate if these provider experience carry any rural-urban distinctions.	Thank you. We did not find stricken differences between the urban and rural divide than the few cases reported, i.e., in lines 342. The conclusion also refers to these insights.
12	D. Discussion<br>1. The discussion does a good job to compare Zambia’s experiences with those of other countries. It would be useful to refer to the key lessons and implications of these findings for policy and practice in Zambia and other countries implementing or considering NHIS.	We thank the reviewer on the empowering insights on policy and practice implications. We attempted to include these in lines across the discussion and in lines 710 and 723.
13	There are usually concerns about NHIS being a ‘trojan horse’ towards	Thank you. These are important reflections on the implication of the inclusion of private

	privatization of health service delivery l there by undermining UHC goals. Private sector inclusion in health service provision is related to some unintended outcomes e.g crowding out public providers due to dysfunctional competition between public and private providers in same settings. Are the private providers in Zambia's NHIS indeed complimentary as suggested? If so, what makes it work? What could be learned from the institutional arrangements in place?	providers in the NHIS. Our study identified the areas on complimentary. But also, the challenges this brought onto the private sector such as overcrowding highlighted in lines 453. These identified are emerging issues that would need additional research to drawn lessons on. Following the reviewers advise, we have included this as an additional area of need for future research in lines 730 and 735.
14	How about the concerns about market failures such as moral hazards specifically supplier-induced demand? What do these mean for the NHIS in Zambia or elsewhere	Thank you. The present article discusses sustainability as part of lines 581 to 647. The policy implications have been included in lines 706 to 712 as advised.
15	The authors could also reflect on the data systems and claims verification processes in private sector: how easy is it to harmonize data systems and what safeguards exist or could be adopted to enrich data quality and curb fraud?	Thank you so much for the suggested inclusions. The section, 'claims' on lines 523 to 577, highlighted the data and information system challenges for the NHIS and discussed in lines 671 to 673. However, as we did not explicitly review the data systems, our discussion was limited to the insights from the study results.

VERSION 2 - REVIEW

Reviewer 1

Name Katz , Aaron

Affiliation University of Washington , Department of Health Services

Date 12-Dec-2024

COI

I appreciate the changes and additions made by the authors. In particular, the Zambia context discussion is very helpful. I would suggest, in lines 225-234, that the authors clarify whether the per capita expenditure data are annual. Also, it would help the reader if the changes in expenditure percentages cited were all for the same two periods (e.g., one change is noted from 2015, another from 2020); perhaps two pie charts (2021 and ?) would make it easier to see the cited changes.

Reviewer 2

Name Ssennyonjo, Aloysius

Affiliation **Makerere University School of Public Health, Health Policy Planning and Management**

Date **11-Jan-2025**

COI

The authors addressed my comments and concerns adequately. I am satisfied with the current version.

VERSION 2 - AUTHOR RESPONSE

Authors Feedback to Reviewer Comments

SN	Comment	Feedback
**Review Comments to the Author**		
**Reviewer #1**		
1	I appreciate the changes and additions made by the authors. In particular, the Zambia context discussion is very helpful.	Thank you for your insightful feedback.
2	I would suggest, in lines 225-234, that the authors clarify whether the per capita expenditure data are annual. Also, it would help the reader if the changes in expenditure percentages cited were all for the same two periods (e.g., one change is noted from 2015, another from 2020); perhaps two pie charts (2021 and ?) would make it easier to see the cited changes.	Thank you for this observation. We have now indicated the current health expenditure data to be for the year 2022.<br><br>The changes in expenditure percentages have been adjusted and now compared for two time points; 2022 and 2020.
**Reviewer #2**		
3	The authors addressed my comments and concerns adequately. I am satisfied with the current version.	The authors, thank the reviewer for their thoughtful feedback for the entire review iterations

---

VERSION 3 - REVIEW

Reviewer **1**

Name Katz , Aaron

Affiliation University of Washington , Department of Health Services

Date 21-Jan-2025

COI

I am satisfied the authors have addressed all reviewers’concerns.
